# The effect of chronic kidney disease on the association of tricuspid regurgitation with overall survival: Insights from SHEBAHEART big data

**DOI:** 10.1007/s40620-025-02377-4

**Published:** 2025-09-15

**Authors:** Ranel Loutati, Viana Copeland, Robert Klempfner, Sagit Ben-Zekry, Efrat Mazor-Dray, Paul Fefer, Israel Moshe Barbash, Victor Guetta, Amit Segev, Rafael Kuperstein, Elad Maor, Pazit Beckerman

**Affiliations:** 1 The Olga and Lev Leviev Heart Center, Chaim Sheba Medical Center Hospital, Tel Hashomer, Israel; 2 School of Medicine, Tel Aviv University, Tel Aviv, Israel; 3 Institute of Nephrology and Hypertension, Sheba Medical Center Hospital-Tel Hashomer, 52621 Ramat Gan, Israel

**Keywords:** Tricuspid regurgitation, Chronic kidney disease, Right ventricle

## Abstract

**Background:**

Chronic kidney disease (CKD) is a common comorbidity among patients with tricuspid regurgitation, yet its impact on tricuspid regurgitation outcomes is underexplored. This study examines how CKD affects the relationship between severe tricuspid regurgitation and overall survival.

**Methods:**

This is a retrospective cohort study of all adult patients (> 18 years old) evaluated at the Sheba Medical Center, between 2007 and 2022, who underwent transthoracic echocardiographic evaluation. It is based on the SHEBAHEART big data registry. Sheba Medical Center is the largest hospital in Israel with approximately 115,000 admissions per year. The echocardiographic reports together with the electronic medical records of all patients are the source for this study. Patients with missing creatinine data within one month of their echocardiography study, as well as those who underwent tricuspid regurgitation intervention, were excluded from the study. Patients were categorized into four groups, according to the presence and severity of tricuspid regurgitation and stratified by CKD stage. The primary outcome was all-cause mortality.

**Results:**

The study included 78,147 patients (median age 67, IQR 55–78), with 2989 (4%) having severe tricuspid regurgitation and 19,910 (25%) with an estimated glomerular filtration rate [eGFR] < 60 mL/min/1.73 m^2^. Over a median 4-year follow-up, 28,112 patients (36%) died. Both tricuspid regurgitation severity and CKD stage were associated with increased mortality risk (log-rank *p* < 0.001 for both). Adjusted models showed that compared to the none/trivial group, patients with mild, moderate, and severe tricuspid regurgitation had a 6%, 12%, and 35% higher risk of death, respectively (*p* < 0.001 for all). The association of tricuspid regurgitation with poor survival was CKD-dependent, with increased mortality risk of 56% vs. 23% among patients with eGFR < 60 vs. eGFR ≥ 60 (*p* for interaction < 0.001). The interaction analysis was no longer significant when right ventricular function was incorporated into the multivariable model. Subanalysis, limited to patients with isolated tricuspid regurgitation, yielded consistent results.

**Conclusions:**

The association between severe tricuspid regurgitation and poor survival is stronger in advanced CKD patients and may be modulated through right ventricular function.

**Graphical abstract:**

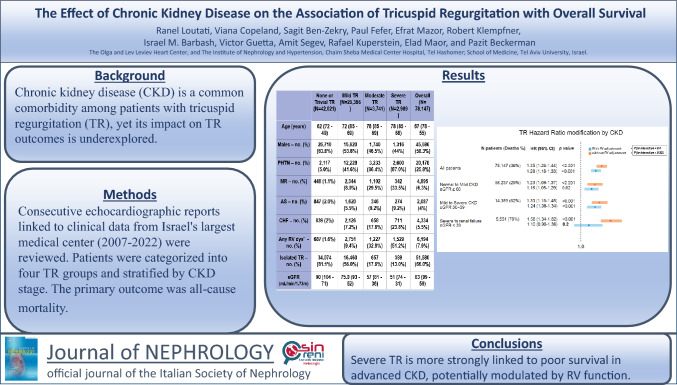

**Supplementary Information:**

The online version contains supplementary material available at 10.1007/s40620-025-02377-4.

## Introduction

Tricuspid regurgitation is a common valvular heart disease, and its prevalence is increasing [1]. The association between tricuspid regurgitation with comorbidities has been widely investigated, and the assertion that the majority of severe tricuspid regurgitation cases are secondary to left heart or pulmonary disease is substantiated by robust clinical data [2, 3]. However, data from registries and retrospective analyses suggest that severe tricuspid regurgitation is independently associated with poor outcome irrespective of other, significant, comorbidities [4–6].

Chronic kidney disease (CKD) is highly prevalent among patients with severe tricuspid regurgitation [7–10]. Numerous studies have explored the link between CKD and tricuspid regurgitation, revealing a mutual connection between the two. Significant tricuspid regurgitation can lead to right-sided heart failure and subsequent kidney dysfunction [8], and concurrently, impaired kidney function is associated with a worse prognosis among patients with tricuspid regurgitation [9, 10]. Despite this established association, CKD patients are under-represented in clinical trials examining transcatheter interventions for tricuspid regurgitation [11, 12], and observational data on the impact of advanced CKD on severe tricuspid regurgitation outcomes are scarce [13].

In Israel, until 2022, transcatheter tricuspid interventions were not reimbursed. This created a unique opportunity to investigate the contemporary outcome of severe tricuspid regurgitation in a large cohort of patients treated with medical therapy alone. Therefore, the objective of this present study was to investigate the effect of CKD on the association of severe tricuspid regurgitation with overall survival using a large and contemporary database, in order to emphasize the prognostic implications of advanced kidney failure among patients with severe tricuspid regurgitation.

## Methods

### Study population

This is a retrospective cohort study of all adult patients (> 18 years old) evaluated at the Sheba Medical Center, between 2007 and 2022, who underwent transthoracic echocardiographic evaluation. It is based on the SHEBAHEART big data registry that was described previously [14]. Sheba Medical Center is the largest hospital in Israel with approximately 115,000 admissions per year. The echocardiographic reports together with the electronic medical records of all patients are the source for this study. Patients with missing creatinine data within one month of their echocardiography study, as well as those who underwent tricuspid regurgitation intervention, were excluded from the study. The Institutional Review Board of the Sheba Medical Center approved this study based on strict maintenance of participants' anonymity during analyses. No individual consent was obtained.

### Standard echocardiographic and Doppler measurements

The Sheba Medical Center echocardiography laboratory is a tertiary laboratory, staffed with academic board-certified cardiologists, all of whom completed echocardiography fellowships. Tricuspid regurgitation was categorized as none, trivial, mild, mild-to-moderate, moderate, moderate-to-severe, and severe using an integrative, semi-quantitative approach. This approach encompassed the assessment of color Doppler jet area, tricuspid valve morphology, right atrium and right ventricle size, inferior vena cava size and respiratory variation, hepatic venous reversal flow, jet density, and contour of continuous wave Doppler envelope, aligning with the guideline recommendations [15, 16]. For this analysis, patients with mild-to-moderate tricuspid regurgitation were pooled with patients with moderate tricuspid regurgitation. Equivocal cases that were coded moderate-to-severe were grouped with severe tricuspid regurgitation cases. Right ventricular size and function were qualitatively assessed on a scale from none to severe using various views, including short-axis parasternal views at basal, mid, and apical levels, lower parasternal right ventricular inflow view, apical 4-chamber view, and right ventricular long-axis and subcostal views when feasible, in accordance with established guidelines [17]. Besides gradation, right ventricular function was assessed quantitatively by tricuspid annular plane systolic excursion measured in the apical four-chamber view, in 12,147 patients. The estimation of right atrial pressure, systolic pulmonary artery pressure, left ventricular diameters, left ventricular volumes, left ventricular ejection fraction (LVEF), and left-sided heart disease were evaluated and measured as recommended [11, 12, 17].

### Definition of isolated tricuspid regurgitation.

 The morphologic type of tricuspid regurgitation known as isolated tricuspid regurgitation is gaining recognition as a distinct entity [18]. Our study includes a subgroup analysis of patients meeting the criteria for isolated tricuspid regurgitation, which requires the absence of pulmonary hypertension and left-sided heart disease, indicated by preserved LVEF, ≤ mild mitral regurgitation, and ≤ mild aortic stenosis.

### Clinical data and study endpoint

Baseline demographic, inpatient status, and clinical data, including laboratory results, comorbidities, and medication use, were obtained from electronic patient records. Diagnoses relied on hospitalization records, laboratory tests, medications, physiological signals (e.g., ECGs), radiological images (e.g., echocardiograms), and procedure reports. Data accuracy, including diagnoses, laboratory results, and medication use, was verified through selected patient case reviews. The estimated glomerular filtration rate (eGFR) was determined using the Chronic Kidney Disease Epidemiology Collaboration creatinine equation. Subgroup analyses according to CKD severity were based on the National Kidney Foundation Kidney Disease Outcomes Quality Initiative staging as follows: normal-mild (stages 1 and 2, eGFR ≥ 60 mL/min/1.73 m^2^), moderate (stage 3, eGFR 30–59 mL/min/1.73 m^2^), and severe to kidney failure (stages 4 and 5, eGFR < 30 mL/min/1.73 m^2^). Proteinuria, measured in mg/g of urine creatinine from a random spot sample within one month of echocardiography, was available for 11,551 patients, categorized as normal/category 1 (< 150 mg/g), category 2 (150–500 mg/g), or category 3 (> 500 mg/g), with subgroup analysis performed accordingly. The primary outcome of the current study was all-cause mortality. Survival data were available for all subjects from the Israeli Population Register up to December 31, 2022.

### Statistical analysis

Continuous variables were expressed as mean ± standard deviation if normally distributed or median with interquartile range if skewed. Categorical variables were presented as frequency (%). Differences between the four tricuspid regurgitation groups were analyzed using one-way ANOVA for continuous variables that were normally distributed, while the Kruskal–Wallis test was used to compare continuous variables that did not adhere to a normal distribution. The inclusion date was the date on which echocardiographic analysis was performed, and follow-up was considered to end on the date of death when applicable or on the date of survival data retrieval (December 31, 2022). The probability of death according to the study groups was graphically displayed using the Kaplan–Meier method, with a comparison of cumulative incidence across strata by the log-rank test. Univariable and multivariable Cox proportional hazards regression modeling were used to compare patients with severe tricuspid regurgitation to patients with none or trivial tricuspid regurgitation, with adjustments made for parameters which were found to be significant in the univariable model or are recognized to impact the survival of patients with valvular disease. The adjusted Cox model incorporated the following variables: age, sex, body mass index, systolic blood pressure, diuretic treatment (including loop diuretics, thiazides, and mineralocorticoid receptor antagonists), clinical diagnosis of heart failure, LVEF, ≥ moderate aortic stenosis and ≥ moderate mitral regurgitation, and systolic pulmonary artery pressure > 40 mmHg, and whether the echocardiographic assessment was performed during hospitalization or in the ambulatory setting. Interaction analysis, using CKD stage as a categorical variable, was used to identify the modification effect of kidney function on the association of severe tricuspid regurgitation with overall survival. To assess whether the effect of CKD was influenced by right ventricular function, the multivariable model was subsequently adjusted by incorporating right ventricular function and comparing interaction significance with and without right ventricular dysfunction. Sensitivity analyses were also conducted to ensure the generalizability of our findings. All analyses were performed using R software version 4.3.3 (R Foundation for Statistical Computing). An association was considered statistically significant for a two-sided P value of less than 0.05.

## Results

### Study population and kidney function

The final study population comprised 78,147 patients, with a median age of 67 (interquartile range [IQR]: 55–78). Of these patients, 45,586 (58%) were men. Most patients had none or trivial tricuspid regurgitation (*n* = 42,021 [54%]), followed by mild tricuspid regurgitation (*n* = 29,396 [38%]), moderate tricuspid regurgitation (*n* = 3741 [5%]), and severe tricuspid regurgitation (*n* = 2989 [4%]) (Supplementary Fig. 1). Overall, 27,308 (35%) patients underwent echocardiographic evaluation during hospitalization, with no significant difference between the four tricuspid regurgitation groups (p = 0.08). Table 1 provides the baseline characteristics of patients stratified by tricuspid regurgitation severity. Patients with severe tricuspid regurgitation were predominantly older women and exhibited higher rates of comorbidities. Compared with those with none or trivial tricuspid regurgitation, patients with severe tricuspid regurgitation were more likely to present with reduced LVEF (34% vs. 8%; *p* < 0.001) and right ventricular dysfunction (51% vs. 2%; *p* < 0.001). Isolated tricuspid regurgitation patients comprised 66% (*n* = 51,580) of the entire study population and 13% (*n* = 389) of the severe tricuspid regurgitation subgroup. Supplementary Fig. 2 depicts the breakdown of tricuspid regurgitation etiologies graphically.

**Table 1:** Baseline characteristics by tricuspid regurgitation severity

	All (*N* = 78,147)	None/trivial TR (*N* = 42,021)	Mild TR (*N* = 29,396)	Moderate TR (*N* = 3741)	Severe TR (*N* = 2989)	*p* Value
Age (median)	67 (55–78)	62 (49–72)	72 (62–81)	78 (69–85)	78 (68–85)	< 0.001
Male, no. (%)	45,586 (58.3%)	26,710 (63.6%)	15,820 (53.8%)	1740 (46.5%)	1316 (44%)	< 0.001
BMI (Kg/m^2) (median)	26.5 (23.7–30.1)	26.7 (23.9–30.3)	26.2 (23.5–29.6)	26.1 (23.3–29.8)	26.1 (23.1–30)	< 0.001
HTN, no. (%)	35,661 (45.6%)	16,049 (38.2%)	15,536 (52.9%)	2271 (60.7)	1805 (60.4%)	< 0.001
DM, no. (%)	20,550 (26.3%)	9877 (23.5%)	8437 (28.7%)	1219 (32.6%)	1017 (34%)	< 0.001
IHD, no. (%)	27,048 (34.6%)	12,693 (30.2%)	11,331 (38.5%)	1708 (45.7%)	1316 (44%)	< 0.001
CHF, no. (%)	4334 (5.5%)	839 (2.0%)	2126 (7.2%)	658 (17.6%)	711 (23.8%)	< 0.001
Atrial fibrillation, no. (%)	16,904 (21.6%)	4516 (10.7%)	8557 (29.1%)	2008 (53.7%)	1823 (61%)	< 0.001
COPD, no. (%)	6071 (7.8%)	2394 (5.7%)	2723 (9.3%)	512 (13.7%)	442 (14.8%)	< 0.001
Malignancy, no. (%)	10,845 (13.9%)	5234 (12.5%)	4645 (15.8%)	554 (14.8%)	412 (13.8%)	< 0.001
Stroke/TIA, no. (%)	14,666 (18.8%)	7092 (16.9%)	6130 (20.9%)	842 (22.5%)	602 (20.1%)	< 0.001
PVD, no. (%)	4722 (6%)	1938 (4.6%)	2130 (7.2%)	359 (9.6%)	295 (9.9%)	< 0.001
Cognitive Impairment, no. (%)	2959 (3.8%)	1137 (2.7%)	1434 (4.9%)	226 (6.0%)	162 (5.4%)	< 0.001
Serum Creatinine (mg/dL)(median)	0.93 (0.74–1.19)	0.89 (0.72–1.08)	0.97 (0.76–1.28)	1.15 (0.84–1.68)	1.27 (0.9–1.88)	< 0.001
eGFR (mL/min/1.73 m^2^) (median)	83 (59–99)	90 (71–104)	75 (52–93)	57 (36–81)	51 (31–74)	< 0.001
Anti coagulations, no. (%)	19,244 (24.6%)	5715 (13.6%)	9413 (32.0%)	2125 (56.8%)	1991 (66.6%)	< 0.001
Beta blockers, no. (%)	30,646 (39.2%)	11,889 (28.3%)	14,204 (48.3%)	2449 (65.5%)	2104 (70.4%)	< 0.001
Diuretics, no. (%)	18,887 (24.2%)	4883 (11.6%)	9529 (32.4%)	2268 (60.6%)	2207 (73.8%)	< 0.001
ACEi/ARB, no. (%)	32,783 (42%)	13,869 (33.0%)	14,566 (49.6%)	2375 (63.5%)	1973 (66%)	< 0.001
SBP (mmHg) (median)	129 (115–145)	129 (115–145)	129 (115–145)	130 (116–147)	130 116–146)	0.00347
DBP (mmHg) (median)	73 (64–81)	73 (64–81)	73 (64–81)	73 (64–82)	73 (65–81)	0.628
Echo performed during admission, no. (%)	27,308 (35%)	14,723 (35%)	10,302 (35%)	1294 (34.6%)	989 (33.1%)	0.08
LVEF < 40%, no. (%)	10,547 (13.5%)	3143 (7.5%)	5138 (17.5%)	1245 (33.3%)	1021 (34.2%)	< 0.001
AS ≥ mod., no. (%)	2087 (4%)	847 (2.0%)	1620 (5.5%)	346 (9.2%)	274 (9.2%)	< 0.001
MR ≥ mod., no. (%)	4895 (6.3%)	448 (1.1%)	2344 (8.0%)	1102 (29.5%)	342 (33.5%)	< 0.001
LVEDD (cm) (median)	4.6 (4.24–5)	4.6 (4.23–5.00)	4.6 (4.25–5.00)	4.6 (4.24–5.00)	4.6 (4.21–5.01)	1
LVESD (cm) (median)	2.9 (3.32 0 2.51)	2.9 (2.51–3.33)	2.9 (3.32–2.5)	2.88 (3.3–2.51)	2.9 (3.3–2.51)	0.475
LVMI (g/m^2^) (median)	85 (70–105)	85 (70–104)	85 (70–105)	85.5 (71–105)	86 (71–106)	0.564
sPAP (mmHg) (median)	33 (28–43)	29 (25–33)	37 (31–46)	54 (45–63)	57 (47–68)	< 0.001
PHTN, no. (%)	20,178 (25.8%)	2117 (5.0%)	12,228 (41.6%)	3233 (86.4%)	2600 (87%)	< 0.001
Any RV dysfunction, no. (%)	6194 (7.9%)	687 (1.6%)	2751 (9.4%)	1227 (32.8%)	1529 (51.2%)	< 0.001
Isolated TR, no. (%)	51,580 (66%)	34,074 (81.1%)	16,460 (56.0%)	657 (17.6%)	389 (13%)	< 0.001
RA area (cm^2^) (median)	14.7 (11.8–18)	14.5 (11.7–18)	14.9 (11.8–18)	15 (12–18)	14.3 (11.9–18)	0.257
RA pressure (mmHg) (median)	10 (5–10)	5 (5–10)	10 (5–10)	10 (5–10)	10 (5–10)	0.0352
Pacemaker, no. (%)	3246 (4.2%)	1721 (4.1%)	1239 (4.2%)	158 (4.2%)	128 (4.3%)	0.963

Values are no. (%) or median (IQR)

*ACEi* angiotensin converting enzyme inhibitors, *ARB* angiotensin receptor blockers, *AS* aortic stenosis, *BMI* body mass index, *CHF* congestive heart failure, *COPD* chronic obstructive pulmonary disease, *DBP* diastolic blood pressure, *DM* diabetes mellitus, *eGFR* estimated glomerular filtration rate, *HTN* hypertension, *IHD* ischemic heart disease, *LVEDD* left ventricular end diastolic diameter, *LVEF* left ventricular ejection fraction, *LVESD* left ventricular end systolic diameter, *LVMI* left ventricular mass index, *MR* mitral regurgitation, *PHTN* pulmonary hypertension, *PVD* peripheral vascular disease, *RV* right ventricle, *RA* right atrium, *SBP* systolic blood pressure, *sPAP* systolic pulmonary arterial pressure, *TIA* transient ischemic attack, *TR* tricuspid regurgitation

At baseline, an eGFR < 60 (all eGFR units are in ml/ minute) was present among 19,910 (25%) patients, with 14,359 (18%) presenting with eGFR between 30–60 and 5551 (7%) with eGFR < 30. Among patients with severe tricuspid regurgitation, *n* = 1144 (38%) had an eGFR ≥ 60, 1172 (39%) had an eGFR between 30–60, and 673 (23%) had an eGFR < 30. In comparison to those with none or trivial tricuspid regurgitation, patients with severe tricuspid regurgitation had significantly lower eGFR (51 [IQR: 31–74] vs. 90 [IQR: 71–104]; *p* < 0.001).

### Tricuspid regurgitation and mortality during follow-up

During a median follow-up period of 4 years (IQR: 1–7), a total of 28,112 (36%) patients died. The crude number of deaths among patients with none/trivial, mild, moderate, and severe tricuspid regurgitation were 10,507 (25%), 13,060 (44%), 2427 (65%), and 2118 (71%), respectively. Kaplan–Meier survival analysis revealed that the cumulative probability of death at 4 years of follow-up was 21% ± 0.2%, 38% ± 0.3%, 59% ± 0.8%, and 70% ± 0.9%, respectively (Supplementary Fig. 3; *p* Log rank < 0.001 for the overall difference during follow-up). Unadjusted Cox model demonstrated that patients with severe tricuspid regurgitation were 5 times more likely to die compared to those with none/trivial tricuspid regurgitation (95% Confidence Interval [CI] 4.80–5.27, *p* < 0.001). Multivariable Cox analysis consistently demonstrated that severe tricuspid regurgitation was associated with a significant and independent 35% increased risk of death during follow-up (95% CI 1.26–1.44, *p* < 0.001). Additionally, Table 2 illustrates other independent predictors of poor survival identified by the multivariable model.

**Table 2:** Multivariable Cox regression analysis

	HR	95% CI	*p* Value
TR severity^a^			
Mild TR	1.06	1.03–1.10	< 0.001
Moderate TR	1.12	1.05–1.19	< 0.001
Severe TR	1.35	1.26–1.44	< 0.001
Age	1.04	1.04–1.04	< 0.001
Sex (male)	1.04	1.01–1.07	0.011
BMI (continuous) for every 5 kg/m^2^	0.92	0.91–0.93	< 0.001
SBP (continuous) for every 20 mmHg	1.02	1.01–1.02	< 0.001
Diuretics	1.38	1.33–1.43	< 0.001
Echo performed during admission	0.99	0.96–1.02	0.6
CHF	1.20	1.15–1.26	< 0.001
Reduced LVEF^b^	1.24	1.19–1.28	< 0.001
Left valvular heart disease^c^	1.19	1.12–1.26	< 0.001
PHTN^d^	1.49	1.43–1.54	< 0.001

*AS* aortic stenosis, *BMI* body mass index, *CHF* congestive heart failure, *CI* confidence interval, *HR* hazard ratio, *LVEF* left ventriclular ejection fraction, *MR* mitral regurgitation, *PHTN* pulmonary hypertension, *SBP* systolic blood pressure, *sPAP* systolic pulmonary artery pressure, *TR* tricuspid regurgitation

^a^None/Trivial TR as the reference group

^b^Reduced LVEF = LVEF ≤ 40%

^c^Left valvular heart disease = at least moderate AS or MR

^d^PHTN = sPAP ≥ 40 mmHg

### Modification effect of CKD on the association of tricuspid regurgitation with survival

**Fig. 1:**
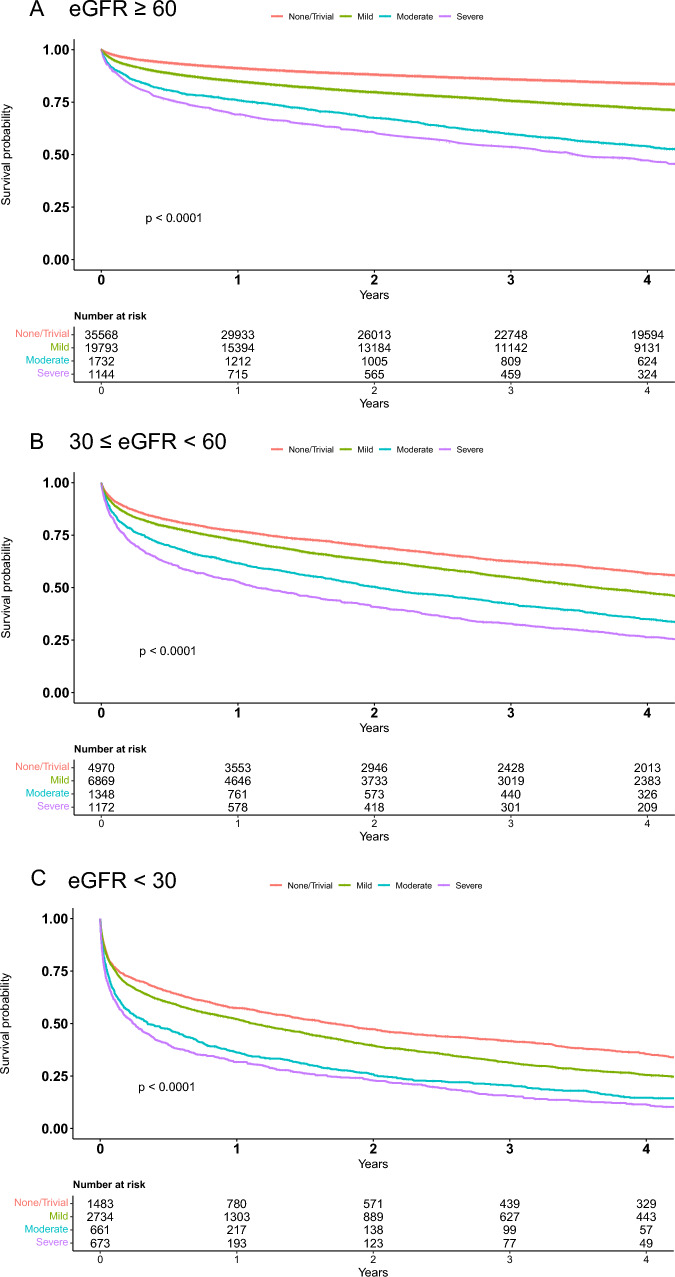
The Kaplan–Meier survival curves exhibiting higher mortality rates with increasing TR severity in all CKD stages. **A** eGFR ≤ 60 (*n* = 58,237), **B** ≥ 30eGFR < 60 (*n* = 14,359), **C** eGFR < 30 (*n* = 5551). Log rank *p* < 0.001 for all curves. *CKD* chronic kidney disease, *eGFR* estimated glomerular filtration rate, *TR* tricuspid regurgitation

**Fig. 2:**
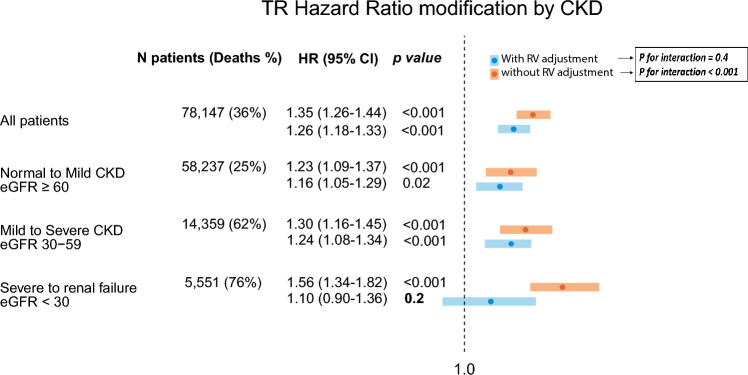
Forest plot of severe TR HR's demonstrates that as kidney function deteriorates, the increased risk of mortality due to severe TR increases (*p* for interaction < 0.001). When incorporating RV dysfunction into the model, HR in patients with eGFR < 30 is no longer significant (*p* for interaction = 0.4). *CKD* chronic kidney disease, *eGFR* estimated glomerular filtration rate, *HR* hazard ratio, *RV* right ventricle, *TR* tricuspid regurgitation

During follow-up, baseline CKD was a substantial predictor of poor survival. The mortality rates among the three pre-specified CKD groups were 25% (14,947/58,237), 62% (8920/14,359), and 77% (4245/5551), respectively. Compared with patients who had eGFR ≥ 60, patients with eGFR < 30 were 6 times more likely to die during follow-up in a univariable model (95% CI 5.67–6.07; *p* < 0.001). Multivariable Cox analysis showed that eGFR < 30 was associated with a significant and independent threefold increased risk of death during follow-up when compared with eGFR ≥ 60 (hazard ratio [HR] 2.8 95% CI 2.6–3, *p* < 0.001). Severe tricuspid regurgitation was found to be associated with excess mortality across all stages of CKD, including patients with eGFR < 30. Kaplan–Meier survival analysis demonstrated that patients with severe tricuspid regurgitation in the three CKD groups had cumulative probability of death at 4 years of 52% ± 1.6% for eGFR ≥ 60, 63% ± 1.4% for eGFR between 30–59, and 90% ± 1.4% for eGFR < 30 (Fig. 1; *p* Log rank < 0.001 for the overall difference during follow‐up). Multivariable Cox-regression with interaction analysis demonstrated a CKD-dependent association between severe tricuspid regurgitation and poor survival. Specifically, in the eGFR ≥ 60 group, severe tricuspid regurgitation was linked with a 23% increase in the risk of death, whereas among patients with eGFR < 60, the risk was more than 2 times higher and reached 56% in the same multivariable model (*p* for interaction < 0.001; Fig. 2).

### Importance of right ventricular function

Multivariable binary logistic regression demonstrated an independent association between CKD and right ventricular dysfunction (*p* < 0.001). Therefore, an additional analysis was performed. The analysis revealed that while there was an excess mortality associated with severe tricuspid regurgitation across all CKD subgroups, and while a significant interaction between CKD stage and tricuspid regurgitation severity existed, the above interaction was no longer significant when the model was further adjusted for right ventricular dysfunction (*p* for interaction = 0.4). Additionally, while severe tricuspid regurgitation was still associated with an increase in the probability of death in patients with eGFR ≥ 30 (HR 1.16 95% CI 1.05–1.29, *p* = 0.02 for eGFR ≥ 60; HR 1.24 95% CI 1.11–1.37, *p* < 0.001 for eGFR between 30–59), in patients with eGFR less than 30, severe tricuspid regurgitation was not associated with excess mortality when right ventricular dysfunction was incorporated into the model (HR 1.10, 95% CI 0.90–1.36, *p* = 0.2; Fig. 4). Tricuspid annular plane systolic excursion measurements were available for 12,147 patients with the following tricuspid regurgitation severity: 5293 (12.6%) with none/trivial tricuspid regurgitation, 5182 (17.6%) with mild tricuspid regurgitation, 855 (23%) with moderate tricuspid regurgitation, and 817 (27.3%) with severe tricuspid regurgitation. A strong correlation was observed between the tricuspid annular plane systolic excursion measurements and visually assessed right ventricular function (Supplementary Fig. 4). The use of tricuspid annular plane systolic excursion in the mediation analysis was consistent with the previous results, whether it was used in a continuous manner (HR 1.18 95% CI 1.10–1.28, *p* = 0.011 for eGFR ≥ 60; HR 1.29 95% CI 0.94–1.75, *p* = 0.084 for eGFR between 30–59; HR 1.36 95% CI 0.91–2.03, *p* = 0.1 for eGFR < 30); *p* for interaction = 0.33), or in a dichotomous manner with cutoff at 1.7 cm (HR 1.22 95% CI 1.10–1.35, *p* = 0.01 for eGFR ≥ 60; HR 1.30 95% CI 0.95–1.75, *p* = 0.074 for eGFR between 30–59; HR 1.34 95% CI 0.91–2.05, *p* = 0.11 for eGFR < 30); *p* for interaction = 0.34).

In an attempt to isolate the effect of the right ventricle, a similar interaction analysis was performed for the right atrial pressure. Further adjustment of the multivariable model for right atrial pressure did not alter the interaction between CKD stage and tricuspid regurgitation severity (p for interaction < 0.001), nor did it alter the association between severe tricuspid regurgitation and mortality across all CKD stages (HR 1.16 95% CI 1.04–1.30, *p* = 0.09 for eGFR ≥ 60; HR 1.34 95% CI 1.22–1.48, *p* < 0.001 for eGFR between 30–59; HR 1.46 95% CI 1.23–1.74, *p* < 0.001 for eGFR < 30).

### Subgroup and sensitivity analyses

Despite smaller sample size, subgroup analysis of isolated tricuspid regurgitation cases demonstrated results that were consistent with the main analysis: the association between severe tricuspid regurgitation and poor survival remained CKD-dependent (*p* for interaction < 0.001). Similar to the main analysis, when right ventricular dysfunction was added to the interaction analysis model, severe tricuspid regurgitation was no longer associated with excess mortality among patients with eGFR < 30 (HR 1.04, 95% CI 0.70–1.52, *p* = 0.8) and the interaction was not significant (*p* for interaction = 0.8; Supplementary Table 1).

Proteinuria, a key marker of kidney and cardio-renal dysfunction, was used as an additional parameter for CKD identification and risk stratification. Among 11,551 patients (15%) with available measurements, 9217 (80%) had normal/category 1 proteinuria (< 150 mg/g), 1791 (15.5%) had category 2 (150–500 mg/g), and 543 (4.5%) had category 3 (> 500 mg/g). Multivariable Cox models yielded results consistent with the main analysis: in category 1, severe tricuspid regurgitation was associated with a 28% increased mortality risk (HR 1.28, 95% CI 1.13–1.42, *p* < 0.001), while in categories 2–3, the risk rose to 40% (HR 1.40, 95% CI 1.21–1.62, *p* < 0.001). When adjusting for right ventricular dysfunction, this association persisted only in category 1 (HR 1.16, 95% CI 1.04–1.29, *p* = 0.01), but not in categories 2–3 (HR 1.27, 95% CI 0.96–1.67, *p* = 0.089). Additional subgroup analyses by a combination of proteinuria levels and CKD stages is given in Supplementary Table 2.

Multiple sensitivity analyses were conducted, separately excluding cases of left heart disease, pulmonary hypertension, and presence of right ventricular leads, and all showed consistent results. After excluding 3246 patients with pacemakers or implantable cardioverter-defibrillators, the association between severe tricuspid regurgitation and mortality remained strong (HR 5.00, 95% CI 4.75–5.25; *p* < 0.001). In patients with eGFR ≥ 60, severe tricuspid regurgitation was associated with a 9% increased risk of death, while in those with eGFR < 60, the risk rose significantly to 29% in the same multivariable model (*p* for interaction < 0.001).

## Discussion

Our analysis offers several important findings. Firstly, it uses contemporary data that confirm and expand previous observations concerning the independent association of severe tricuspid regurgitation with poor survival [2, 3]. Secondly, it demonstrates how this association is CKD-dependent, such that the excess mortality associated with tricuspid regurgitation increases as kidney function worsens. Finally, this analysis provides an original perspective into tricuspid regurgitation and CKD interrelation, highlighting the role of right ventricular function as a possible mediator between tricuspid regurgitation, CKD, and overall survival.

Severe tricuspid regurgitation is associated with many comorbidities. Anand et al. [10] employed unsupervised clustering analysis to identify distinctive subgroups among tricuspid regurgitation patients, and showed how CKD patients had the worst outcome. The Tricuspid Regurgitation Impact on Outcomes (TRIO) score has previously been proposed as a validated and straightforward clinical risk score for predicting mortality in patients with significant tricuspid regurgitation [19]. The main link between tricuspid regurgitation and the clinical variables used in calculating the score is attributed to systemic venous congestion. Our analysis substantiates this observation by demonstrating that the significance of the interaction between CKD stage and tricuspid regurgitation severity, and the association between severe tricuspid regurgitation and excess mortality, are diminished upon inclusion of right ventricular dysfunction, which serves as a driving force for increased systemic venous congestion, in our model.

While CKD is known to be associated with worse outcome among cardiovascular patients [20, 21], this is the first large analysis to examine the potential modifying effect of CKD in the specific case of severe tricuspid regurgitation. In a prior investigation involving 444 individuals with tricuspid regurgitation, Schipmann et al. [22] demonstrated that CKD progression is associated with increased risk of mortality. Samad et al. retrospectively investigated 78,059 patients, 30% of whom had CKD. They found higher rates of left-sided valve disease in CKD patients, which was linked to increased mortality [23]. Our results are in line with these studies, suggesting that despite possible competing risk of death due to kidney disease, severe tricuspid regurgitation is associated with excess mortality.

Our analysis suggests a potential mediating role of right ventricular dysfunction on the association between tricuspid regurgitation, CKD and survival. Butcher et al. [9] reviewed 1234 patients with significant tricuspid regurgitation grouped by kidney function. The authors concluded that the link between tricuspid regurgitation and CKD was due to right ventricular dysfunction. A similar inference was drawn in a cohort of 413 patients with chronic systolic heart failure [24]. Our data extend these findings. The results of our study could potentially be explained by the interconnecting pathways between the heart and the kidneys. In the context of tricuspid regurgitation, kidney function can be negatively impacted through two mechanisms: firstly, by decreasing cardiac output, leading to renal hypoperfusion; and secondly, by increasing central venous pressure, causing renal venous hypertension. This process may contribute to a cascade of adverse effects, including inflammation, cytokine release, accelerated atherosclerosis, and CKD-associated cardiomyopathy, characterized by extensive interstitial myocardial fibrosis and structural cardiac changes such as left ventricular hypertrophy and valvular abnormalities, including tricuspid regurgitation [25]. The prognostic impact of right ventricular function in individuals with tricuspid regurgitation has been firmly established [26, 27]. Our group has recently demonstrated how right ventricular function modulates the outcome of severe tricuspid regurgitation [14] by showing that as right ventricular function declines, the difference in outcome between patients with and without severe tricuspid regurgitation becomes less distinct. The aforementioned studies, combined with our current analysis, offer a plausible explanation for the link between tricuspid regurgitation and CKD while indicating the potential mediating role of the right ventricle.

The emergence of transcatheter tricuspid valve interventions has redirected attention towards the challenge of patient selection within the heterogeneous population of individuals with tricuspid regurgitation. Our study demonstrates that the prognostic implications of tricuspid regurgitation become more pronounced as kidney function deteriorates, with this association diminishing in patients with right ventricular dysfunction, pointing to a potentially under-represented subgroup that may benefit from timely management and early intervention. However, given the design limitations of our study, these results should not be directly applied to clinical practice. Focused research is essential to refine selection criteria and improve outcomes for this heterogeneous high-risk patient population.

This study has strengths and limitations. Its major strength is the contemporary data and the fact that in Israel, as of 2022, transcatheter tricuspid interventions are no longer reimbursed. Therefore, all patients included in this cohort were treated medically. This study has several important limitations. First, this is a retrospective, single-center study with all its inherent limitations including referral bias. Second, our study population consisted of all-comers who underwent echocardiographic evaluation at our institute, resulting in a heterogeneous cohort in terms of clinical presentation and comorbidities. While this limits the specificity of our findings to particular subgroups, it enhances the generalizability of our results. Third, cause-specific mortality was not available. However, in Israel, cardiovascular death is a leading cause of death second only to cancer [28]. Fourth, important clinical and laboratory predictors of poor survival such as clinical signs of right heart failure, functional class, and B-type natriuretic peptide were not available. Lastly, since our cohort dates back to 2007, tricuspid regurgitation was not assessed punctiliously in every patient. Hence, quantitative indices of tricuspid regurgitation severity and right ventricular function such as S`, as well as data regarding cardioversion and dialysis, were not readily available for most patients and were not included in the analysis. Finally, while independent associations have been demonstrated, causality could not be established due to study design.

## Conclusions and clinical implications

Severe tricuspid regurgitation is independently associated with excess mortality. In our cohort, this association appeared more pronounced among patients with advanced CKD, underscoring the need for comprehensive CKD status assessment among individuals with severe tricuspid regurgitation. The unfavorable outcome observed in this subgroup may be partly explained by concomitant right ventricular dysfunction.

## Supplementary Information

Below is the link to the electronic supplementary material.

Supplementary file1 (DOCX 294 KB)

## Data Availability

The data underlying this article cannot be shared publicly for the privacy of individuals that participated in the study. The data will be shared on reasonable request to the corresponding author (P.B).
