# Supplementary material for: The effect of chronic kidney disease on the association of tricuspid regurgitation with overall survival: Insights from SHEBAHEART big data
Source: J Nephrol. 2025 Sep 15;38(9):2785–95. doi: 10.1007/s40620-025-02377-4 (PMC12712064; doi:10.1007/s40620-025-02377-4)
Supplement: Supplementary file 1 — Supplementary file1 (DOCX 294 KB) [file 40620_2025_2377_MOESM1_ESM.docx]

Supplementary Material for Manuscript:

**The Effect of Chronic Kidney Disease on the Association of Tricuspid Regurgitation with Overall Survival**

Supplementary Table 1 – Isolated TR subgroup analysis showing hazard ratio (HR) for mortality of severe TR with and without RV dysfunction adjustment across all three CKD stages:

| **P value** | **95% CI** | **HR with RV dys’ adjustment** | **P value** | **95% CI** | **Adjusted HR (for TR)** | **Events (N [%])** | **Patients (N [%])** | **CKD stage**  mL/min/1.73m2 |
| --- | --- | --- | --- | --- | --- | --- | --- | --- |
| 0.6 | 0.83-1.45 | 1.09 | 0.03 | 1.03-1.65 | 1.30 | 9135 (21%) | 42,944 (83%) | eGFR ≥ 60 |
| 0.4 | 0.93-2.2 | 1.11 | <0.001 | 1.57-2.45 | 1.95 | 3592 (53.5%) | 6715 (13%) | eGFR 30-59 |
| 0.8 | 0.69-1.51 | 1.04 | <0.001 | 1.7-3.3 | 2.5 | 1247 (65%) | 1921 (4%) | eGFR < 30 |

CKD = Chronic Kidney Disease; CI = Confidence Interval; HR = Hazard ratio; eGFR = estimated Glomerular Filtration Rate; RV = Right Ventricle

Supplementary Table 2 –Subgroup analysis by proteinuria levels showing hazard ratio (HR) for mortality of severe TR with and without RV dysfunction adjustment across all three CKD stages:

| **CKD stage** mL/min/1.73m2 | **Proteinuria levels** | **HR (95% CI; p-value) without RV dys' adjustment** | **HR (95% CI; p-value) with RV dys' adjustment** |
| --- | --- | --- | --- |
|  | <150 mg/g | 1.07 (0.82-1.27; p=0.9) | 0.97 (0.74-1.27; p=0.8) |
| eGFR ≥ 60 | 150-500 mg/g | 2.05 (1.11-3.79; p=0.022) | 1.96 (1.05-3.67; p=0.035) |
|  | >500 mg/g | 1.28 (0.35-4.67; p=0.7) | 1.12 (0.30-4.19; p=0.9) |
|  | <150 mg/g | 1.40 (1.10-1.79; p=0.007) | 1.29 (1.00-1.66; p=0.052) |
| eGFR 30-59 | 150-500 mg/g | 0.97 (0.53-1.76; p>0.9) | 0.85 (0.46-1.57; p=0.6) |
|  | >500 mg/g | 5.23 (1.48-18.5; p=0.01) | 3.21 (0.78-13.2; p=0.11) |
|  | <150 mg/g | 1.81 (1.31-2.50; p<0.001) | 1.61 (1.15-2.24; p=0.005) |
| eGFR < 30 | 150-500 mg/g | 1.54 (0.98-2.44; p=0.064) | 1.38 (0.86-2.22; p=0.2) |
|  | >500 mg/g | 0.93 (0.38-2.25; p-0.9) | 0.75 (0.30-1.91; p=0.5) |

CKD = Chronic Kidney Disease; CI = Confidence Interval; HR = Hazard ratio; eGFR = estimated Glomerular Filtration Rate; RV = Right Ventricle


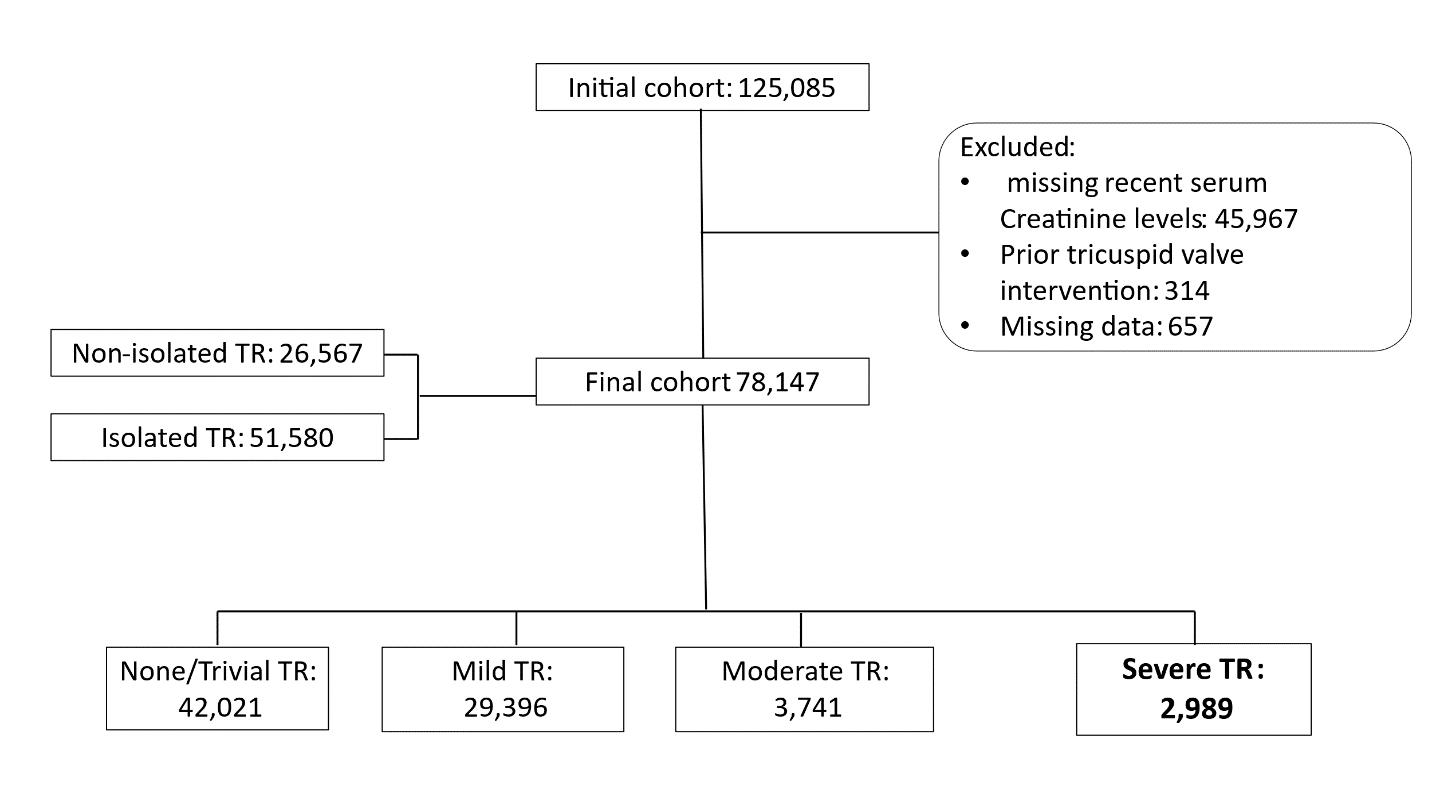
Supplementary Figure 1 – Study flow chart:

Supplementary Figure 1 legend:

The flow of participants showing the number of patients screened and excluded.
TR = Tricuspid Regurgitation.

Supplementary Figure 2 – Bubble plot of TR etiologies by TR severity:


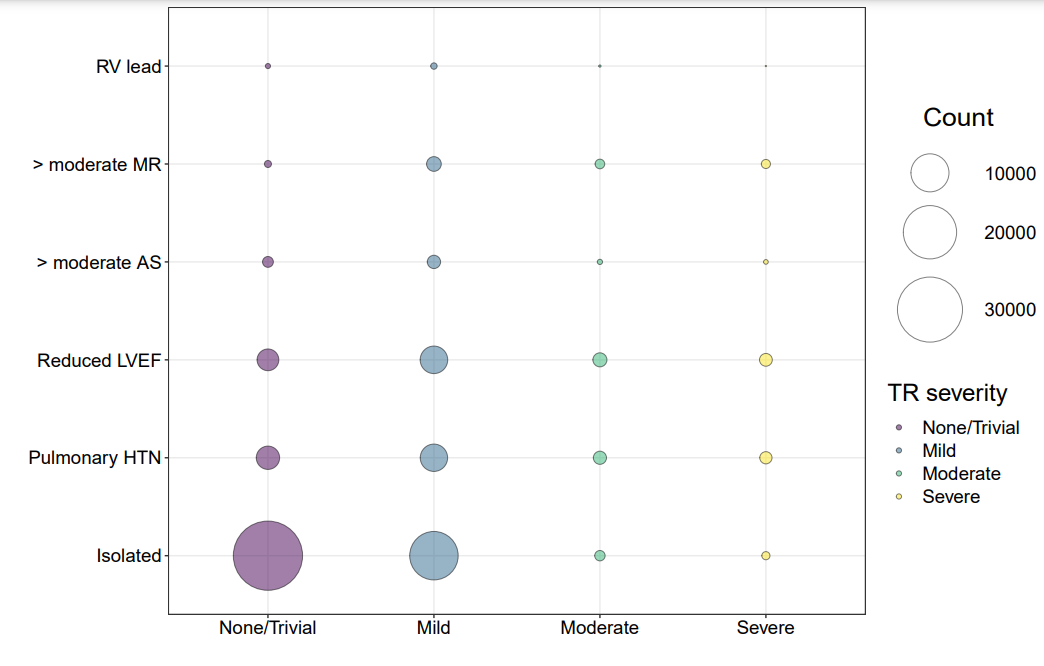


Supplementary Figure 2 legend:

This plot depicts the distribution of patients across TR severity groups, stratified by the principal etiologies of TR. Categories were not mutually exclusive, as some patients had several coexisting pathologies. AS = Aortic Stenosis. HTN = Hypertension. LVEF = Left Ventricular Ejection fraction. MR = Mitral Regurgitation. RV = Right Ventricle. TR = Tricuspid Regurgitation.

Supplementary Figure 3 - Kaplan-Meier survival analysis by TR severity without CKD stratification:


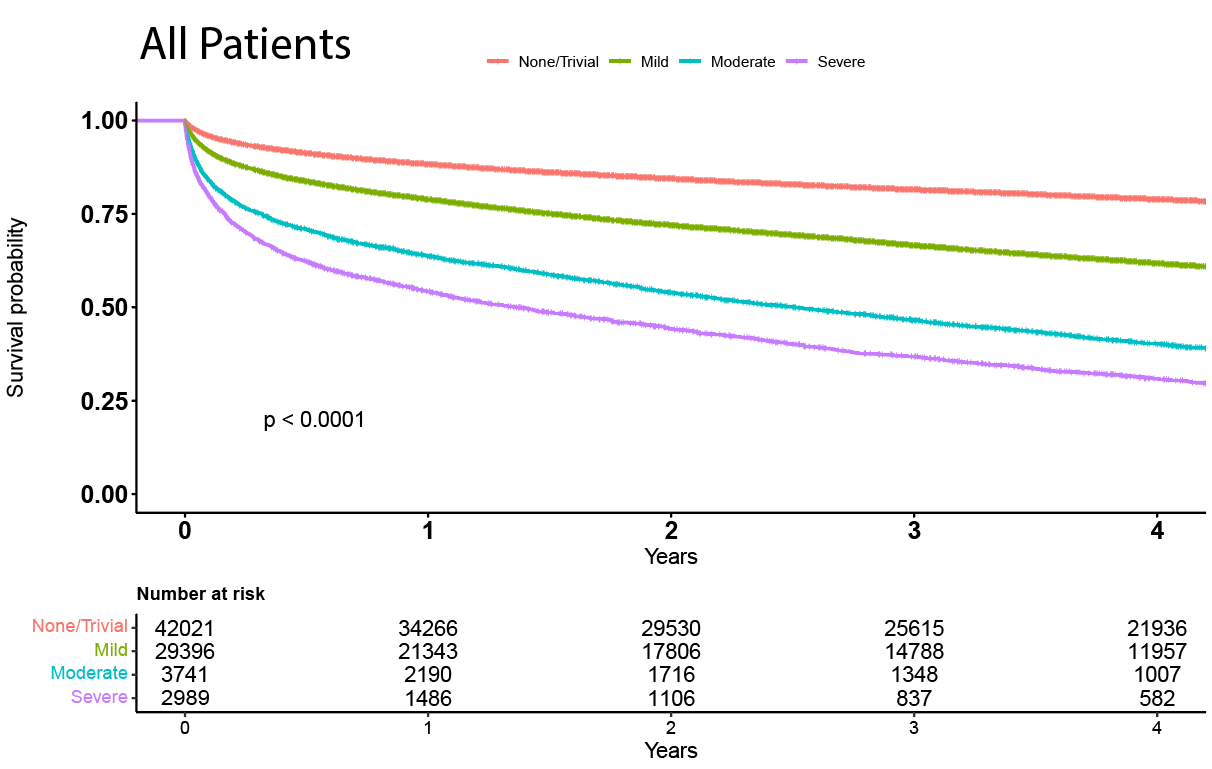


Supplementary Figure 3 legend:

The Kaplan-Meier survival curves demonstrating higher mortality rates with increasing TR severity in all patients (n=78,147). Log rank p < .001. CKD = Chronic Kidney Disease. TR = Tricuspid Regurgitation.

Supplementary Figure 4 – Box plot of TAPSE values in visually assessed RV function:


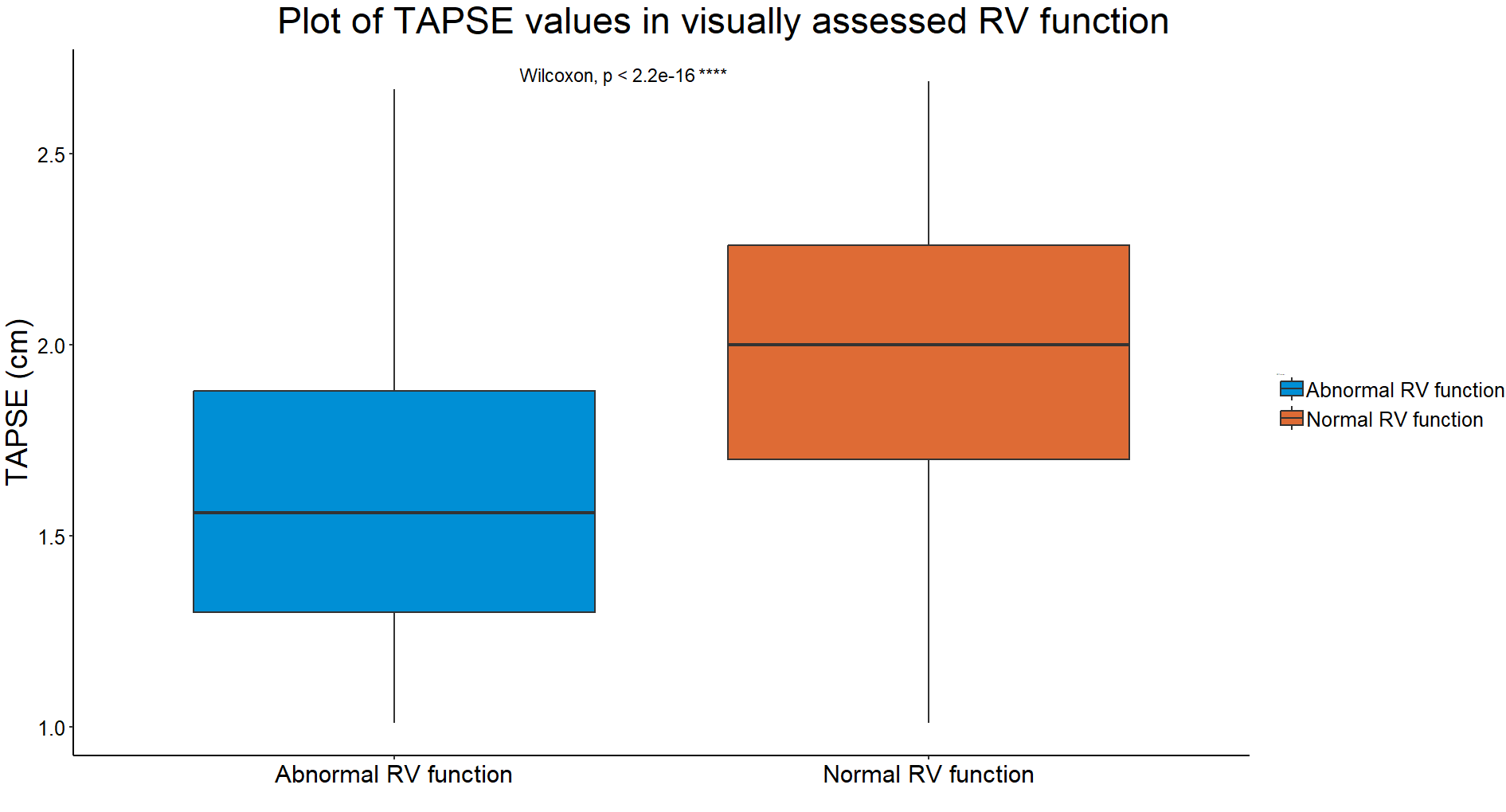


Supplementary Figure 4 legend:

TAPSE values and visually assessed RV function demonstrate an excellent correlation, which validates the use of visual assessment for RV function.
RV = Right Ventricle. TAPSE = Tricuspid Annular Plane Systolic Excursion.
